# Enhancing Industrial Communication with Ethernet/Internet Protocol: A Study and Analysis of Real-Time Cooperative Robot Communication and Automation via Transmission Control Protocol/Internet Protocol

**DOI:** 10.3390/s23208580

**Published:** 2023-10-19

**Authors:** JuYong Seong, Rahul Ranjan, Joongeup Kye, Seungjae Lee, Sungchul Lee

**Affiliations:** 1 Division of Computer Science and Engineering, Sunmoon University, Asan 31460, Republic of Korea; humphery27@sunmoon.ac.kr (J.S.); rahulranjan@sunmoon.ac.kr (R.R.); leeko@sunmoon.ac.kr (S.L.); 2 Department of Mechanical Engineering, Intelligent Robot Research Institute, Asan 31460, Republic of Korea; jekye@sunmoon.ac.kr

**Keywords:** Ethernet/IP, common industrial protocol, TCP/IP, virtual simulator, programmable logic controllers, explicit messaging, industrial communication network

## Abstract

This study explores the important task of validating data exchange between a control box, a Programmable Logic Controller (PLC), and a robot in an industrial setting. To achieve this, we adopt a unique approach utilizing both a virtual PLC simulator and an actual PLC device. We introduce an innovative industrial communication module to facilitate the efficient collection and storage of data among these interconnected entities. The main aim of this inquiry is to examine the implementation of Ethernet/IP (EIP), a relatively new addition to the industrial network scenery. It was designed using ODVA’s Common Industrial Protocol (CIP™). The Costumed real-time data communication module was programmed in C++ for the Linux Debian platform and elegantly demonstrates the impressive versatility of EIP as a means for effective data transfer in an industrial environment. The study’s findings provide valuable insights into Ethernet/IP’s functionalities and capabilities in industrial networks, bringing attention to its possible applications in industrial robotics. By connecting theoretical knowledge and practical implementation, this research makes a significant contribution to the continued development of industrial communication systems, ultimately improving the efficiency and effectiveness of automation processes.

## 1. Introduction

Recent developments in the field of robotics and robotization have increased diligence, enhancing productivity and efficiency [1]. As demand for sophisticated robotics and robotization grows, the reliability and efficiency of communication systems linking these machines become crucial [2]. Therefore, it is imperative to design advanced communication modules and protocols for optimal data collection and storage from robotic systems [3,4]. Improving the connectivity and data transfer capabilities of industrial networks, with a focus on Ethernet/IP (EIP), is crucial. These refinements guarantee uninterrupted communication between robotic systems and data storage infrastructures. Additionally, it is urgent to establish a standardized application layer that guarantees real-time control and universal interoperability over Ethernet TCP/IP [5]. A diverse range of networks and Ethernet technologies have been utilized in the manufacturing industry over the last decade. Ethernet, having become the standard in various other fields, including the Internet, is now an attractive alternative in this industry [6]. The heightened interest in the topic has been further increased due to the reduction in bias costs and the faster network speeds [7,8]. The contributions of this study are summarized as follows:

1. Our study undertakes a rigorous verification of data exchange mechanisms involving a control box, a Programmable Logic Controller (PLC), and a robotic system. This comprehensive evaluation forms the foundation of our research.
2. We bridge the gap between theoretical simulations and real-world applications by integrating a virtual PLC simulation platform and an actual PLC apparatus. This integration enhances the practicality and relevance of our findings.
3. An innovative industrial communication module designed specifically to facilitate seamless data aggregation and archival is introduced. This module represents a significant technological advancement, providing a valuable solution for industrial automation.
4. We present the verification of data transmission, including event and packet verification, utilizing an EIP simulator and EIP(XGL EFMTB) [9]. Through these tests, we provide empirical evidence of the reliability and robustness of the developed modules, which has practical implications for the field of industrial automation.

This study demonstrates the effectiveness and trust ability of the developed communication modules and protocols, which can be useful in the development of advanced robotics and robotization systems.

In our paper, we follow a structured approach to address the research problem. In the “Related Work” section, we provide an overview of the existing literature on data communication between a control box, a Programmable Logic Controller (PLC), and a robot, laying the foundation for our study. The “Methodology” section details our unique approach, which incorporates both a virtual PLC simulator and our proposed frame structure for physical communication verification. We also utilize PLC EIP(XGL EFMTB) and client protocols in our methodology. Moving to the “Proposed Work” section, we elaborate on the frame structure and its role in enhancing data transmission and decoding processes. In the “Result and Discussion” section, we present and analyze the outcomes of our study, particularly focusing on the verification of data transmission and the decoding of LS Elc.’s Frame Format. Finally, in the Conclusion, we summarize our key findings and emphasize their significance in advancing the realm of robotics and automation systems whilst suggesting potential directions for future research.

## 2. Related Work

Numerous studies have analyzed industrial networks, with a specific emphasis on Ethernet/IP. They have emphasized the significance of a standardized application layer that allows for interoperability, ensuring universal compatibility and real-time control across Ethernet TCP/IP [5]. A fresh design has been developed for EIP Intelligent Communication Modules (EIICM) [10] utilized in industrial Programmable Logic Controllers (PLCs). The EIICM serves as a network communication module, a CPU for the operation switch, and provides messaging services. The design encompasses a comprehensive architecture featuring fundamental techniques such as “Structured Message Broker” and “Management-self Adaptable” patterns. The EIICM system facilitates communication with diverse devices, conforms to the ODVA [11] specification, governs the switch, and enables configuration and diagnosis. With concurrency and synchronization, the system supports EIP and Mod-bus/TCP protocols, real-time data transfer, and considerable workloads [12].

The creation of a dependable and adaptable EIP adapter for cooperative robots increases their scalability and ability to transfer data effectively. This module is both reliable and flexible, enabling communication system development time to be reduced [13]. Ethernet- and IP-based routing are critical for the future of Electric Vehicle (EV) communication networks [14]. Ethernet provides a cost-effective and flexible solution for efficient data transmission. Its powerful capacity enables high-speed data transfer, which contributes to the seamless operation of modern communication networks. Ethernet-based architectures expedite standardization, leading to cost-effective development and a plethora of compatible products. The utilization of Ethernet as the fundamental structure permits communication between vehicles and infrastructure (V2I) [15] and between vehicles (V2V) [16], integrating vehicular networks with Dedicated Short-Range Communication (DSRC) [17], Wireless Access in Vehicular Environments (WAVE) [18], and charging infrastructure.

Automotive Ethernet offers key benefits in conjunction with the ongoing use of Controller Area Network (CAN) [19] and FlexRay [20] for safety and body domains [21]. Packet devices that use network processors and traffic management processors can produce flows with assigned bandwidths [22]. The performance of the EIP network, one of the top Real-time Ethernet networks in factory automation systems, is exemplary. The study examines two Performance Indicators, Delivery Time and Throughput in Real-time Ethernet (RTE), shedding light on unpredictable delays that affect practical data. Further investigations will explore more complex architectures and additional Performance Indicators [23]. The development of a PC/Ethernet-based Fieldbus Network for Large Real-time Data Communication is also discussed [24].

This paper covers a range of topics related to industrial networks, such as the necessity of standardized Application Layers for interoperability, the creation of effective EIP adapters for cooperative robots, multimedia-based learning modules for vocational education, and performance analysis of EIP networks.

## 3. Work Methodology

In this section, we present a systematic approach to achieving data communication among a control box, a Programmable Logic Controller (PLC), and a robot utilizing a virtual PLC simulator. We detail the selection of tools and components, configuration of communication protocols, and setup of specific data exchange scenarios. Our methodology includes data collection, analysis, and the implementation of repeatable experiments to ensure the robustness of our findings. Ethical considerations related to the experiments are also briefly addressed. This section provides a clear and structured framework for understanding how we conducted our research, ensuring the credibility and reliability of our results.

### Data Communication among a Control Device, a PLC, and a Robot Using a Virtual PLC Simulator

The control device and robot were created referencing the ODVA EIP technical documentation. The programming language implemented is C/C++, while the operating system chosen is Linux Debian.

TCP/IP is a suite of communication protocols that enable data transmission over the Internet and other networks. TCP/IP is widely used in various industrial applications, such as remote monitoring, control, automation, and data acquisition. However, TCP/IP alone does not specify how the data should be structured, encoded, or exchanged between different devices and systems. Therefore, various application-layer protocols have been developed to provide specific functionalities and features for different industrial use cases. Some of the most common and popular protocols that use TCP/IP are MQTT, Modbus TCP, PROFINET, and OPC-UA. In this section, we will briefly introduce these protocols and compare their advantages and disadvantages in the context of Industry 4.0 and Industrial Internet of Things (IIoT). MQTT (Message Queuing Telemetry Transport) is a lightweight and simple publish–subscribe protocol that enables efficient data transmission between devices and systems over low-bandwidth and unreliable networks [25]. MQTT uses a broker to manage communication between publishers (data sources) and subscribers (data consumers). Publishers send messages to topics, which are logical channels that categorize the data. Subscribers subscribe to topics of interest and receive messages from the broker. MQTT supports three levels of quality of service (QoS) to ensure reliable message delivery: once at the most, at least once, and exactly once. MQTT also supports features such as retained messages, last will and testament, and keep-alive mechanism to enhance communication efficiency and robustness [26].

**Figure 1:**
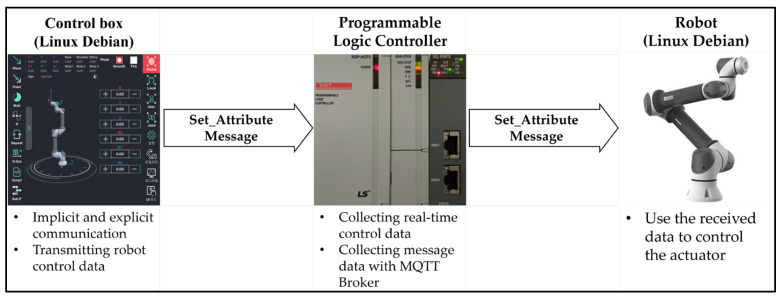
Using EIP for sending and receiving explicit messages and device interactions.

**Figure 2:**
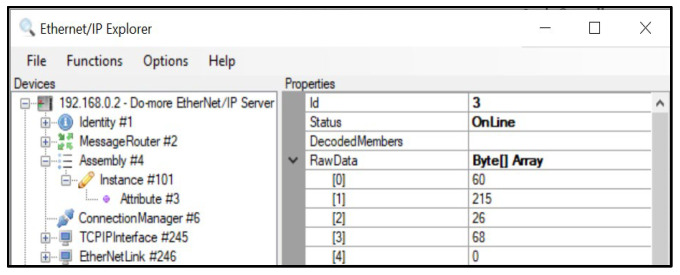
Verifying data fetching from PLC simulator using the EIP explore tool.

1. The communication between the control device and the robot was tested with a PLC simulator before conducting experiments with a live PLC. Technical abbreviation will be explained upon first use.
2. Figure 1 shows the direct message process utilizing an industrial communication module that enables communication between the control device (Rainbow Robotics Ether-Net/IP module), the PLC simulator, and the robot. The main aim is to ascertain the dependability of communication between the devices and evaluate the effectiveness of data collection and storage procedures.
3. The communication process starts with the control device transmitting a “Set Attribute” command to the PLC simulator.
4. The PLC simulator involves sending a memory payload with randomly generated numbers ranging from 0 to 255. The second stage involves monitoring memory changes. To assess the communication process, we utilize the EIP explorer tool to keep track of alterations in the memory values of the PLC simulator. Specifically, we monitor changes in memory locations 0, 1, 2, and 3 within category 4, instance 101, and attribute 3, as depicted in Figure 2.
5. Subsequently, the PLC simulator sends a “Set-Attribute” command to the Rainbow Robotics virtual simulator and robots by Rainbow Robotics to transmit data to the robot. This instruction entails the transmission of eight bytes of data, spanning from V20 to V23, as designated in Table 1.
6. The robot receives the “Set_Attribute” command, processes it, and finally generates output results.

A simulation was carried out to confirm data transmission between the control device and the Do-more PLC simulator [27]. A random number within the 0–255 range was generated and sent from the control device to the PLC simulator. Figure 2 displays the raw data in a message packet from the Do-more PLC simulator following the transmission of the ‘Set_Attribute’ command from the control box. The EIP explorer tool 1.2 [28] was employed. This software application was created to examine the contents of the PLC simulator’s memory. The tool facilitates the identification of EIP devices that are linked to the local network and the ability to view or edit the memory of those devices.

**Figure 3:**
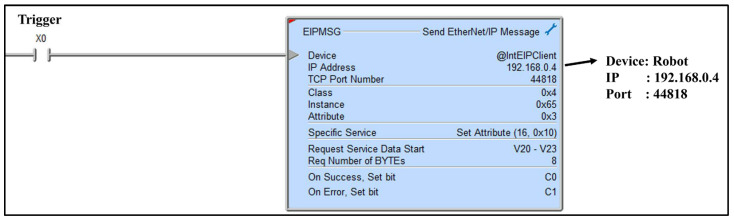
Transmitting data using the ‘Set-Attribute’ method in Ladder Logic.

To ensure the integrity and coherence of information exchanged between the control box and the PLC simulator, a meticulous examination of the raw data residing within the memory of the PLC simulator is imperative. Employing the λ (lambda) simulation mechanism, meticulously scrutinize the process of data transmission and reception transpiring between the PLC simulator and the robot, an emulation platform operating on the Linux Debian system. In Figure 3, the device is configured as Linux Debian, representing the robot. The IP and port details are evident. The ‘Set_Attribute’ command facilitates the transmission of data from addresses V20 to V23 stored in the PLC, utilizing the Ladder Logic programming language. This procedure outlines the PLC-to-Robot communication, wherein the PLC forwards values, previously modified by the control box, to the robot. Facilitate the seamless transmission of data originating from the control box, residing within the PLC simulator, towards the robot (192.168.0.4:44818) by employing the “Set_Attribute” functionality within the context of sending Ethernet/IP message.

Configure the “Set_Attribute” option by specifying the precise IP address and port number associated with the robot, ensuring an accurate destination for the variables V20 through V23 derived from the control box. This directed transfer aims to validate the efficacy of data transmission by effectively relaying the variables to the robot, as illustrated in Table 1.

**Table 1:** Memory data from Do-more PLC simulator transmitted to robot.

Index	Element	Status
0	V20	60
1	V21	215
2	V22	26
3	V23	68

**Figure 4:**
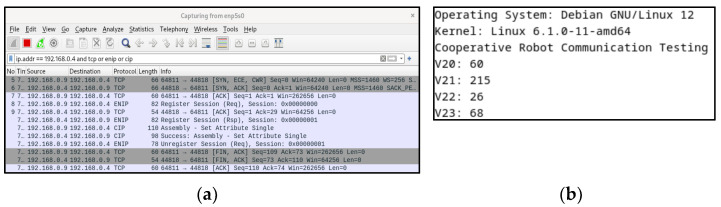
(**a**) TCP, ENIP, and CIP packets verified using WireShark; (**b**) output of the robot receiving the results sent by the PLC simulator.

Figure 4 presents the outcome of transmitting data from V20~V23 memory of PLC simulator to robot through the “Set_Attribute” message. This process facilitated us in ensuring the dependability of the data transmission and reception amid the control box, PLC simulator, and robot. Furthermore, it allowed for real-time control and storage and collection.

## 4. Proposed Structure

Physical Communication Verification: PLC EIP(XGL EFMTB) and clients, we delve into the validation of our communication system’s physical layer. This involves meticulous testing and verification of the PLC EIP protocol, specifically focusing on the XGL EFMTB protocol and its interaction with client devices. Furthermore, in the subsequent section, “Decoding LS Elc.’s Frame Format”, we shift our focus to the data frame format used within the communication process. Here, we provide detailed insights into the decoding process of LS Elc.’s Frame Format, unraveling the intricacies of how data packets are structured and interpreted. These sections collectively form the foundation for evaluating the reliability and integrity of our communication modules and protocols.

### 4.1. Physical Communication Verification: PLC EIP (XGL EFMTB) and Clients

**Figure 5:**
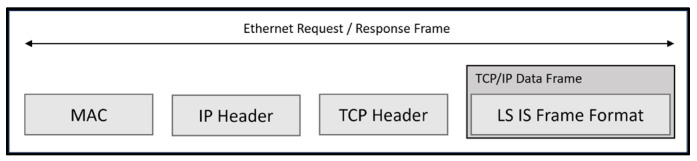
LS IS. EIP frame structure.

To apply the previously verified communication in real-world settings, we substituted the simulator with a physical PLC device to confirm the communication between the control box, simulator, and robot. The physical PLC device utilized LS Elc.’s EIP communication module, XGL EFMTB. As a result, the control device and robot were converted to LS Elc.’s exclusive protocol, and the communication was validated. Figure 5 illustrates the shift from PLC simulation to the application of physical PLC. Initial research was conducted on the data frame for implementing the Programmable Logic Controller (PLC) with LS Elc. The frame is prefixed with a TCP/IP header, followed by a data frame.

To ensure a smooth and reliable exchange of data from the control device to the PLC Ethernet/IP, LS Elc.’s [29] communication frame, conformant with the prescribed communication protocol, is employed. This guarantees a robust and seamless data exchange mechanism. The Media Access Control (MAC) identification number functions as a distinctive identifier for Ethernet devices, allowing them to be distinguished efficiently by their individual properties. The IP header is located at the beginning of an Internet Protocol packet and provides vital information, including IP version, total header length, packet length, source address, destination address, and other details. This header plays a crucial role in enabling effective routing and delivery of data throughout the IP network. The TCP header is a key protocol that ensures a dependable, sequential, and error-free exchange of information between devices. TCP includes a verification mechanism that confirms the precise reception of data by the recipient. While this may result in slightly slower transmission speeds, it ensures the completeness and integrity of the transmitted data.

**Figure 6:**
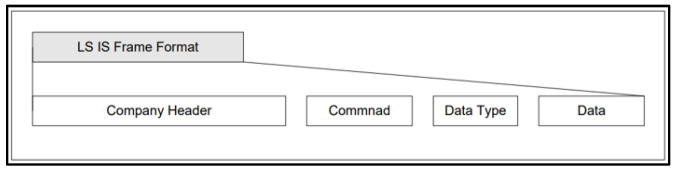
XGT proprietary frame structure.

The control device uses the EIP Stack and LS Elc. frame format to transfer data to the PLC, allowing for real-time collection and storage of received data. The TCP/IP data frame is consistent with the LS Elc. standard frame format, which is a standardized structure that facilitates seamless communication with Elc’s equipment. It ensures compatibility and efficient data exchange when communicating with Elc’s devices. By understanding the attributes and features of these components, a comprehensive comprehension of EIP communication and its corresponding frame structure can be obtained, as shown in Figure 6, which displays LS Elc.’s exclusive XGT communication frame construction for exchanging data with their physical equipment (PLC). The LSIS data communication frame comprises LS ELECTRIC’s exclusive data (Company ID), Command, Data Type, and Data. The frame design is depicted in Figure 5. Specifics of each frame element are shown in Table 2 (Company Header) and Table 3 (Command, Data Type).

### 4.2. Decoding LS Elc.’s Frame Format

The LS IS frame format’s company header segment contains crucial data, providing information about LS Elc. company, specific PLC equipment, module specifications, and other relevant particulars. Technical terms’ abbreviations will be explained when first used. The command field is divided into two unique commands: reading the memory value of PLC EIP equipment and writing values to the memory. The language is objective, value-neutral, and free from bias, keeping a formal register. The text follows conventional academic structure and style, adhering to the requirements of spelling, grammar, and formatting in British English. The data type component includes several data types, including Bit, Byte, Word, DWord, and LWord. Word, as a processing unit, enables the CPU to handle 16 bits at the same time. Double word, or DWord, emerged with the development of CPU performance, enabling it to process up to 32 bits at once. Long word, or LWord, can process 64 bits, double the capacity of DWord. By understanding the complexities of LS Elc.’s frame format, which includes the company header, command, data type, and related data structures, a comprehensive grasp of the communication protocol and data manipulation abilities can be attained. In Table 2, the company header designates a unique machine type identified by the conversion of “LSIS-XGTs” into hexadecimal values followed by NULL characters [30]. This hexadecimal value serves as the identifier for the machine owned by Sun Moon University.

When transmitting data from a personal computer (PC) acting as a client to a PLC utilizing PLC EIP as a server, the PLC Info field should be populated with 0x00. Alternatively, if using PLC EIP as a server, it is necessary to specify the pertinent information regarding the current PLC, including details such as CPU type, redundancy, CPU error troubleshooting, and others. When transmitting frames from a PC client to a PLC EIP server, it is appropriate to use 0x33 as the client-to-server source indicator. Each frame that is transmitted is assigned a unique identifier by the Invoke ID to avoid any confusion for multiple frames. The length field indicates the total length of the command, data type, and data area.

The Ethernet location in which the EIP module is installed should also be noted. For research and development purposes, slot 0 was utilized, with a value of 0x00 inputted. The reserved area designates the section allocated for frame formatting by LS Electric (LS Elc.), which ensures optimal frame organization [30].

There are four commands used by the XGT-specific protocol, each of which handles read/write, request/response. The available data types for each command are bits, bytes, words, doublewords, and longwords when discrete, and bytes only when contiguous [31].

The Programmable Logic Controller’s (PLC) memory was accessed and altered from the control device through a write request frame. As per Table 4, the write command function directly specifies the device memory and its data type. Up to 16 independent device memories can be written simultaneously.

Table 5 presents an instance of a write command formed by combining Table 2, Table 3 and Table 4. This showcases the creation of a frame that includes the write request command, the word data type, the reservation, the number of blocks, the length of the variable, the name of the variable (the Word type of the M variable, 100), the size of the data, and the data to be written to individual memory.

The company header is appended with command, data type, and data components to form a unified packet to be transmitted. An illustration in the program shows a command aimed at assigning a value to a variable. The program employs two types of write commands: individual write and continuous write. An individual write permits up to 2 bytes to be written, whereas a continuous write permits up to 1400 bytes. Using the aforementioned program, let us formulate and verify programs for individual and consecutive writes, following the ensuing instructions: 1. Execute the command to write the data value ‘1’ to variable D at position 0. 2. Write the data value “Hello World” to variable D at position 0. It is crucial to note that the particular implementation of these programs may differ depending on the programming language, platform, and tools utilized. See Figure 6 for reference.

**Figure 7:**
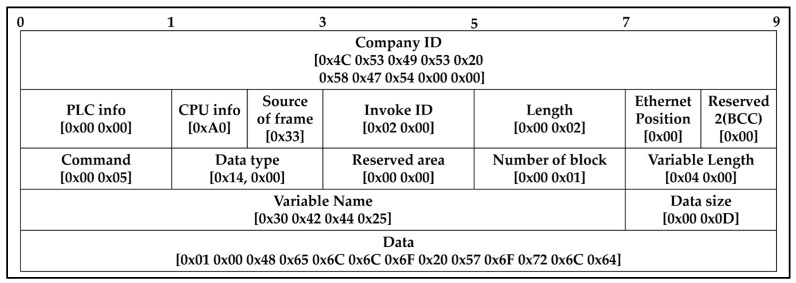
Example of transmitting the prepared write command communication frame to the PLC EIP module for write command.

Figure 7 depicts an illustration of a command packet transmitted through a PLC EIP module. Specifically, this example exhibits how an ASCII code is formed by amalgamating the frames outlined in Table 2, Table 3 and Table 4 to form a comprehensive code. The ensuing code section is provided below.

**Figure 8:**
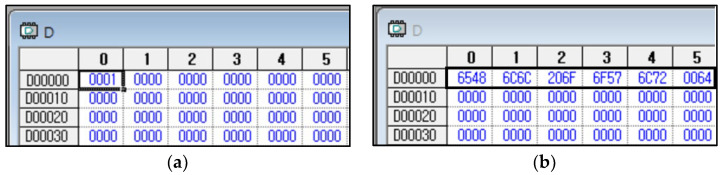
Example of a memory block in a PLC viewed through the XG5000 4.7.2 software: (**a**) Individual writing a value of 1 to block D0; (**b**) Continuous write from block D0 to block n: writing “Hello World”.

**Figure 9:**
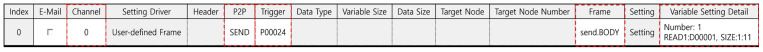
Creating a PLC EIP block.

**Figure 10:**
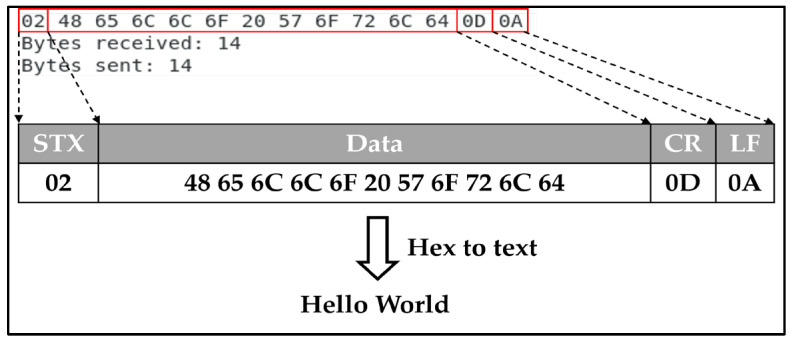
Verifying data reception on the robot (Linux Debian).

Verifying the obtained outcomes using the XG5000 4.7.2 [33], the software tool 4.7.2 tailored for designing and debugging the XGT PLC series. Figure 8a,b depicts the results. Utilize an individual write mechanism to handle up to 2 bytes for writing a value of ‘1’ to block D0. It is crucial to validate the precision of the write function employing LS Electric’s device monitor. Write the value “Hello World” from block D0 to block n using a contiguous write that can write up to 1400 bytes. After writing, verify with LS Elc.’s device monitor that the value is in hexadecimal format. In Figure 8b, the hexadecimal value corresponds to the “Hello World” text. This example demonstrates how to issue a write command from the control device to the PLC to adjust a particular memory location within the PLC. When this memory location is changed to a value of ‘1’, it triggers the PLC’s mechanism and executes the instruction block depicted in Figure 9. This block then sends the message ‘Hello World’ to the robot, as displayed in Figure 10. Sending the complete frame via a transmitting and receiving program written in C.

## 5. Results

In this section, we provide a comprehensive analysis of three key dimensions. First, we delve into the results of our data transfer experiments, carefully examining metrics such as data transfer rates, latency, and error rates. This analysis provides valuable insights into the efficiency and reliability of data transfer within the LS Electric system [10,34]. Secondly, we examine the intricate workings of the trigger activation and coil control mechanisms within the system, shedding light on their responsiveness and their key role in ensuring seamless and efficient communication between the various system components. Finally, we detail the measures implemented to maintain data integrity and content accuracy during transmission. These safeguards have a significant impact on overall system performance, underlining the critical importance of accurate and error-free data within the LS Electric system.

### 5.1. Verification of Data Transmission

To establish communication between a PLC using EIP protocol and a Linux Debian client, it is necessary to follow certain steps in a professional manner. Firstly, Point-to-Point (P2P) communication should be implemented [33]. This involves establishing a communication setup between the PLC and the Linux Debian client in order to facilitate data transmission. Configuration of network settings and ensuring that both devices are connected to the same network segment is crucial for this setup. Then, a data frame must be created to facilitate the smooth transmission of data. Defining and organizing the data frame, which contains the information to be transmitted from the PLC to the Linux Debian client, is essential. The data frame should cover the necessary variables, tags, or parameters for transmission 3.

Provide the IP address and port of the Linux Debian client. The communication configuration settings of the Programmable Logic Controller (PLC) should clearly state the IP address and port number for the Linux Debian client. This allows the PLC to establish a connection with the designated client and directly transmitted data to the correct destination. When following these steps, a dependable and effective communication channel can be established between the PLC, which uses the EIP protocol, and the Linux Debian client. This enables smooth data transmission by utilizing P2P communication, defining a suitable data frame, and specifying the exact IP address and port of the Linux Debian client. To guarantee success, three frames—HEAD, TAIL, and BODY—are required. The HEAD and TAIL frames possess vital packet information, whereas the BODY frame carries customizable user data.

### 5.2. Protocol and Frame Components for User Data Transmission

There are two protocol types: the standard STD protocol and the SUM protocol, which includes a checksum to the standard variety. The selection of the protocol type is determined by a parameter in the temperature controller. The STD protocol begins with the start character STX (0x02) and concludes with the end characters CR (0x0D) and LF (0x0A), and it is the default protocol included in the HEAD for user frame definitions. The user frame protocol is widely used, with the TAIL section of each frame featuring a carriage return (CR) and line feed (LF) to signal the end of data and mark the beginning of a new data frame. This guarantees proper formatting of the protocol and accurate receipt on the receiving device. Table 6 illustrates the inclusion of CR and LF in the TAIL of the frame. Table 7 outlines the composition of a segment, with the header (HEAD) containing the STX value of 02 and the footer (TAIL) containing the CR and LF values of 0D and 0A, respectively. The body of the segment consists of 11 bytes of data. Consequently, the transmitted data of the segment is made up of 1 byte for the header, 2 bytes for the footer, and 11 bytes for the body, resulting in a total of 14 bytes.

To guarantee the successful delivery of the custom frame, it is crucial to register the IP address and port number of the receiving destination, that is, Linux Debian, with an IP address of 192.168.0.26 and a port number of 8888. Upon registration of these details, the custom frame will be sent to the destination. The blocks serve the purpose of identifying the optimal recipient for a particular segment. One block is tasked with reading 11 bytes from variable D 1. This occurs upon fulfilling the startup condition and then transmitting the segment to recipient 0, who is the Linux Debian client. Refer to Figure 9 for a clear summary of the main points: 1.

The term “channel” pertains to the target receiver and denotes the Linux Debian client in particular. 2. Channel selection determines if the segment should be received or transmitted by the Linux Debian client 3. The starting condition acts as a trigger for the mentioned blocks to commence their operations. The term “frame”, as the data format utilized for transmitting information to the receiver, was previously defined in Section 4. In this particular example, it pertains to a segment consisting of 14 bytes that has been produced. Section 5 outlines the “variable setting content”, which designates the initial memory location for the variable from where the data will be transmitted. In this instance, data will be transmitted from the initial address to the 11th byte, as demonstrated.

### 5.3. Trigger Activation and Coil Control in the LS Electric System

1. The trigger condition is initiated when the value 1 is assigned to the D variable 0.
2. The term “<Coil>(P00024)”, as shown in Figure 9, denotes a coil function that exists on the LS Electric side. This particular coil function performs the action of switching on and off within a specific duration when the trigger condition has been reached.
3. The aforementioned program is intended to trigger the block’s startup condition by activating the <Coil> when the D variable 0 is assigned a value of 1. The aforementioned program is intended to trigger the block’s startup condition by activating the <Coil> when the D variable 0 is assigned a value of 1. The following points provide a more professional summary: 1.
4. The aforementioned program is intended to trigger the block’s startup condition by activating the <Coil> when the D variable 0 is assigned a value of 1.
5. The block’s startup condition is activated when the D variable 0 is assigned a value of 1. 2. “<Coil>” denotes a coil function located on the LS Electric side.
6. The LS Electric side turns on and off for a specific duration when the trigger condition is met. 3. The program is intended to activate the block’s start-up condition by turning on the “<Coil>” when there is a value of 1 assigned to D variable 0.
7. After the value 1 is written to block D0, the coil switches to the “On” position. Furthermore, the bit value of P00024 changes to alternate between on and off states for a duration determined by the user.

### 5.4. Ensuring Data Integrity and Content Accuracy

In order to confirm the successful transmission of the 14-byte segment created by the user-defined frame on the Linux Debian Client, it is essential to verify that the STX 02 value for the HEAD and the CR and LF values of 0D and 0A, respectively, for the TAIL, are received at both positions. The BODY, situated between the HEAD and TAIL, serves to confirm the receipt of the value. The hexadecimal code within the BODY section confirms the transmission and receipt of the phrase “Hello World” displayed in Figure 10.

## 6. Conclusions and Discussion

In this research, our discussion highlights the pivotal role played by the simulator in verifying data transmission across various components, including the PLC EIP simulator and Rainbow Robotics’ virtual simulator. This thorough testing phase has been instrumental in validating communication protocols and ensuring the seamless flow of data among these critical elements.

Furthermore, we emphasize the importance of the Linux Debian and Windows client programs developed using C/C++. These programs have allowed us to perform comprehensive testing, confirming the physical communication of PLC EIP(XGL EFMTB) and TCP/IP with the clients. This practical validation has reinforced the robustness and reliability of the communication protocols and their compatibility with designated clients. Our use of C/C++ programs for data transmission and reception has enabled meticulous analysis of headers and custom packet formation for PLC Ethernet/IP. This has facilitated in-depth scrutiny of the data transmission process, ensuring precise and efficient communication between devices. In summary, our research has leveraged simulators, C/C++ programs, and the development of data transmission and reception programs to successfully verify and validate EIP communication in PLCs. In future work, the simulation of communication using other TCP-based protocols could be an interesting avenue for exploration. In future work, we see opportunities for further refinement and expansion. Leveraging the simulator and C/C++ programs, we will explore advanced functionalities and conduct more intricate testing scenarios. Additionally, we will consider the integration of emerging technologies and protocols to enhance the capabilities of PLC EIP communication. By building on the foundations laid in this study, future research will contribute to the continuous improvement of reliability, compatibility, and overall performance in the field of industrial communication systems.

## Figures and Tables

**Table 2:** Header structure for XGT-only protocols.

Field Name	Field Size (Byte)	Field Value	
Company ID	10	“LSIS-XGT” + “NULL NULL(Reserved) (ASCII CODE: 4C 53 49 53 20 58 47 54 00 00) “LGIS-GLOFA” (ASCII CODE: 4C 47 49 53 20 47 4C 4F 46 41)	
PLC Info	2	Client ➔ Server: Don’t care(0x00) Server ➔ Client: Bit00-05: CPU Type (XGK/I/R-CPUH: 0x01. XGK/I-CPUS: 0x02, XGK-CPUA: 0x03. XGK/I-CPUE: 0x04. XGK/I-CPUU: 0x05, XGK-CPUHN: 0x11. XGK-CPUSN: Ox12, XGI-CPUUN: 0x15) Bit06: 0 (Duplexing Master), 1 (Duplexing Slave) Bit07: 0 (CPU Run), 1(CPU Error) Bit08~12: System State (RUN: 0x01. STOP: 0x02. ERROR: Ox04. DEBUG:0x08) Bit13~15: Reserved	
CPU Info	1	Determine that it is an XGK/XGI/XGR series.	
		XGK: 0xA0 XGI: 0xA4 XGR: 0xA8	XGB(MK): 0xB0 XGB(IEC): 0xB4
Source of Frame	1	Client (HMI, Human Machine Interface) → Server (PLC): 0x33 Server (PLC) ➔ Client (HMI): 0x11	
Invoke ID	2	ID to distinguish the order between frames. (Send this number in the response frame)	
Length	2	The byte size of the command structure	
Ethernet Position	1	Bit0-3: Slot number of the Ethernet module Bits4-7: Base number of the Ethernet module	
Reserved 2(BCC)	1	0x00: Reserved Area (Byte Sum of Header)	

**Table 3:** XGT-specific protocol commands and command codes.

Command	Command Code	Data Type			Description
Write	Request: h’ 0058	Individual	h’ 0000	BIT	Request to write data for each data type
			h’ 0100	BYTE	
			h’ 0200	WORD	
			h’ 0300	DWORD	
			h’ 0400	LWORD	
		Continuous	h’ 1400	BYTE	Request to write a byte variable in blocks

**Table 4:** Frame structure of write commands among XGT specific protocol commands and command codes.

Field Name	Field Size (Byte)	Field Value
Command	2	0x0058: Request to write
Data Type	2	See the data type table [32]
Reserved Area	2	-
Number of blocks	2	The number of variables you want to write to, up to 16
Variable Length	2	Maximum of 16 characters for the length of the direct variable
Variable Name	Variable Length	Only direct variables can be used
Data Size	2	Byte size of the data
Data	Data Size	The data you want to write
…	…	Repeat as many times as variables/max 16 characters
Variable Length	2	Maximum of 16 characters for the length of the direct variable
Variable Name	Variable Length	Only direct variables can be used
Data Size	2	Byte size of the data
Data	Data Size	The data you want to write

**Table 5:** Request frame example for a write command.

Frame Name	Header	Command	Data Type	Reversed	Number of Blocks	Variable Length	Variable Name	Data Size	Data
Code (Example)	..	h’ 0058	h’ 0002	h’ 0000	h’ 0002	h’ 0006	%MW100	h’ 0002	h’ 1234

**Table 6:** Start of Text (STX) standard protocol.

STX	Node Number	Command	Data	Carriage Return	Line Feed
0x02	1~99	-	-	0x0D	0x0A

**Table 7:** Adding segments (HEAD, TAIL, BODY).

-	FRAME DATA				
HEAD (1)	HEAD_00				
TAIL (2)	TAIL_00	TAIL_01			
BODY (14)	HEAD	SEGMENT_00	TAIL		

## Data Availability

Not applicable.
